# Summer crisis in Iran: increase in reported cases of Crimean-Congo Hemorrhagic Fever (CCHF) 

**DOI:** 10.3205/dgkh000303

**Published:** 2017-11-20

**Authors:** Mina Aghamali, Hossein Samadi Kafil

**Affiliations:** 1 Hematology and Oncology Research Center, Tabriz University of Medical Sciences, Tabriz, Iran; 2 Student Research Committee, Tabriz University of Medical Sciences, Tabriz, Iran; 3 Drug Applied Research Center, Tabriz University of Medical Sciences, Tabriz, Iran

## Letter to the Editor

First described as a clinical entity in 1994–1995, Crimean-Congo Hemorrhagic Fever (CCHF) is currently present in many regions of Africa, Asia, the Middle East, and Europe [1]. Annually, >1000 reported CCHF cases result in fatality rates ranging from 10% to as high as 50% in endemic regions [2]. 

The history of CCHF in Iran goes back to 1970, when 45 of 100 sheep sera sent from Tehran to Moscow were positive for CCHF [3]. Thereafter, until 1974–1975, no human infection case was confirmed, although a number of suspected cases had been found previously. Since the first outbreak in Iran in 1999, CCHF has been a major public health concern, demonstrating its endemicity in Iran [4]. It has been reported in 26 of 31 provinces, mostly described in Sistan and Baluchistan, Isfahan, Fars, Tehran, Khorasan, and Khuzestan provinces [5]. In May 2017, a new outbreak of this disease was recorded. First, 19 workers at a slaughterhouse in Sistan and Baluchistan were found to be infected. Thereafter, other cases from Isfahan, Yazd, Kerman, Kermanshah, Hormozgan and Khorasan, and Mazandaran (2 infectious cases) were added to the list. So far, published data by the Iranian Ministry of Health reveals 3 deaths with 33 cases of infection, although the precise number of suspected individuals is not known. It has been claimed that fatalities were associated with animal husbandry and late recognition of disease. Even though the source of this outbreak remains obscure, it is assumed that illegal imports of infected livestock from Afghanistan accounted for the outbreak of CCHF in Sistan and Baluchistan. 

In an effort to confine infection, the government began monitoring exposed individuals, checking the health of livestock before slaughtering, as well as increasing of public awareness in term of personal protective measures. However, the outbreak has not yet been contained. The issue has become the predominant subject of Iranian databases, trying to inform people about probable hazards. To quote a local website presenting concerns about the ongoing situation, “CCHF got the title of most horrible disease of the year”. The increasing number of infected cases has intensified public fear, to the extent that people prefer not to purchase meat products, even those confirmed by a veterinary organization. The unstable price of meat in Iranian markets is the other consequence of the CCHF outbreak. In some regions, people are charged too much by wholesalers due to the outbreak. The other concern is the probable impact of CCHF in an upcoming event, Eid-al-Adha. On this religious occasion, millions of livestock are sacrificed all over the country. As we know, local people in suburbs and rural areas are rarely familiar with guidelines on proper slaughtering; therefore, infections like CCHF, if neglected, could have catastrophic consequences. Limitations related to accurate diagnosis and prolonged confirmation of disease by reference laboratories further exacerbate the matter. The countrywide employment of rapid and applicable approaches to disease prevention and identification seems to be a useful aid for saving many lives. In order to battle the ongoing disease, eradication of unreliable sources of meat in conjunction with early treatment of human patients are necessary. Healthcare workers should strictly follow standard hygience precautions to avert the human-to-human transmission and nosocomial outbreak. Overall, as our neighboring countries – including Afghanistan, Pakistan, and Turkey – are also endemic for CCHF, particularly high risk regions are in serious danger for further outbreaks if prevention planning and prompt control programs are not provided.

## Notes

### Competing interests

The authors declare that they have no competing interests.
